# Elastic modulus evolution of rocks under heating–cooling cycles

**DOI:** 10.1038/s41598-020-70920-3

**Published:** 2020-08-14

**Authors:** Weidong Liu, Liangchi Zhang, Ning Luo

**Affiliations:** 1 Laboratory for Precision and Nano Processing Technologies, School of Mechanical and Manufacturing Engineering, University of New South Wales, Sydney, NSW 2052 Australia; 2 Department of Mechanics and Aerospace Engineering, Southern University of Science and Technology, Shenzhen, 518055 Guangdong China; 3 State Key Laboratory for Geo-Mechanics and Deep Underground Engineering, School of Mechanics and Civil Engineering, China University of Mining and Technology, Xuzhou, 221116 China

**Keywords:** Mechanical engineering, Mechanical properties

## Abstract

Rocks decay significantly during or after heating–cooling cycles, which can in turn lead to hazards such as landslide and stone building collapse. Nevertheless, the deterioration mechanisms are unclear. This paper presents a simple and reliable method to explore the mechanical property evolutions of representative sandstones during heating–cooling cycles. It was found that rock decay takes place in both heating and cooling processes, and dramatic modulus changes occurred near the *α *− *β* phase transition temperature of quartz. Our analysis also revealed that the rock decay is mainly attributed to the internal cracking. The underlying mechanism is the heterogeneous thermal deformation of mineral grains and the *α *– *β* phase transition of quartz.

## Introduction

The mechanical properties of rocks are significantly affected by heating–cooling cycles ^1–7^, which in turn can dramatically increase the risk of landslide^8^, rockfall^2^ and stone building collapse^9^ during or after fire-related accidents. This has been evidenced by many landslide events after the extreme 2003 wildfire in the southern interior of British Columbia (Canada) ^10^, and by the fire-induced loss of about one historic stone building in the European Union each day^11^. Revealing rock deterioration mechanisms has become critical for developing fire rescue scheme and for establishing criteria of post-fire hazard assessment. Furthermore, deep understanding of the rock deterioration mechanisms is the key to conducting thermally assisted excavation/drilling in mining engineering^12^, to the extraction of geothermal energy^13^, and to evaluating the geological conditions in geosciences^2,14^.

Some studies on the property changes of heat-treated rocks have been reported, mainly focusing on the density, porosity, permeability, compressional wave velocity, strength and modulus^4–6,15–18^. The types of rocks studied include sandstones^4–6,19^, granite^15–17^, Marble^6^ and limestone^6,18^. It was found that when the heat treatment temperature *T*_ht_ is below 250 °C, the physical and mechanical property changes are very small^4–6,15–18^. However, if *T*_ht_ is greater than 250 °C, significant rock deterioration can occur and new cracks and pores can be observed in post-heat treatment rocks^4–6,15–18^. It was suggested that the α − β phase transformation of quartz would have contributed to the deterioration of quartz-rich rocks^20,21^. In these studies, however, most of the property and structure characterizations were conducted either before or after a heat treatment. Quantitative characterization of the deterioration process and the effects of thermal expansion/shrinkage and the *α *– *β* transition of quartz are not available. It is unclear how an individual factor influences the mechanical properties of a rock—the key knowledge-base for developing fire rescue schemes and for establishing reliable criteria of post-fire hazard assessment.

Young’s modulus is an important mechanical property measure of rocks, and has been widely used in rock engineering design^3^. The Young’s modulus of a rock can decrease significantly after a heat treatment^4–6,15–18^, which is mainly due to internal microcracking activities^22,23^. As such it can be used as an effective indicator of the rock deterioration. However, traditional methods for modulus measurement, such as uniaxial compression and bending tests, are destructive. They are difficult to be conducted at high temperature, not to mention a continuous measurement with changing temperatures. We found that the high-temperature impulse excitation technique (HTIET) is capable of measuring the elastic properties of advanced materials and their evolution with temperature^24,25^. The advantages of this technique are in its solid theoretical background of vibration, simple set-up, and non-destructive nature. The technique has been applied successfully in characterizing the internal structure and property changes of many different type of materials such as glassy carbons^26,27^, borosilicate glass^28^, and polymer glass^29^.

**Figure 1:**
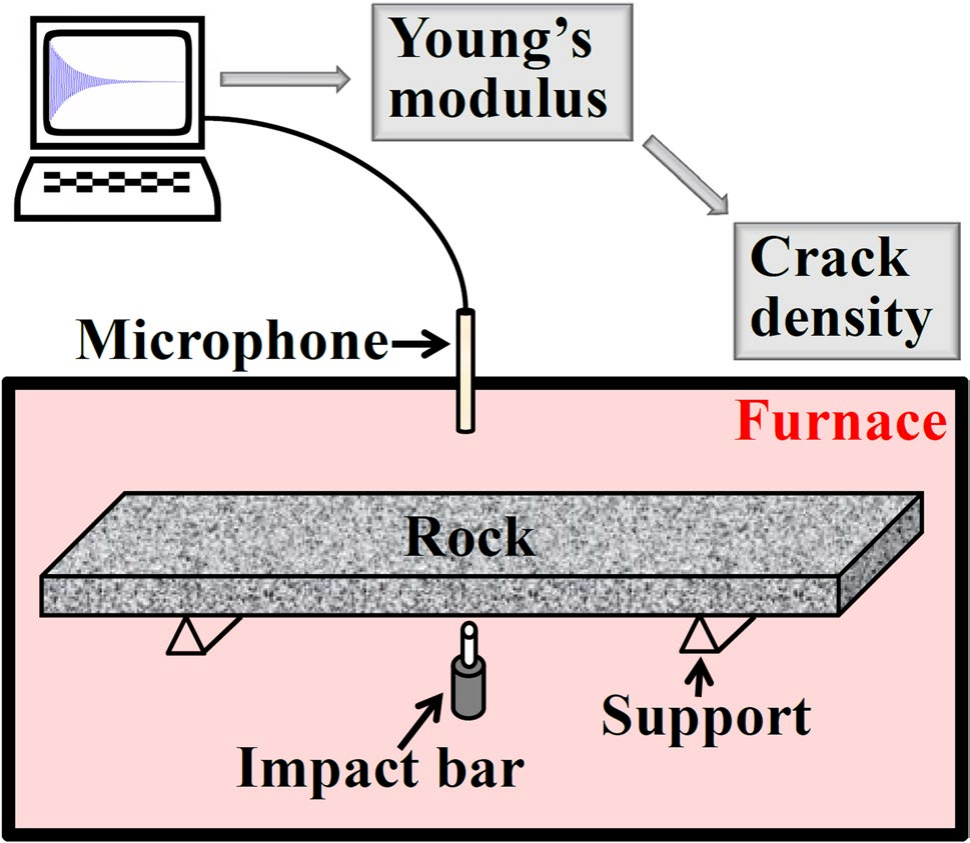
Schematic set-up for a high-temperature impulse excitation test.

This paper aims to reveal the decay mechanisms of representative sandstones during cyclic heating–cooling treatments. To this end, the Young’s modulus evolutions of the sandstones with temperature will be characterized with the aid of the HTIET (see Fig. 1). The underlying deterioration mechanisms will be revealed via a mean-field model linking the modulus evolution with the crack density change in rocks.

## Results

**Figure 2:**
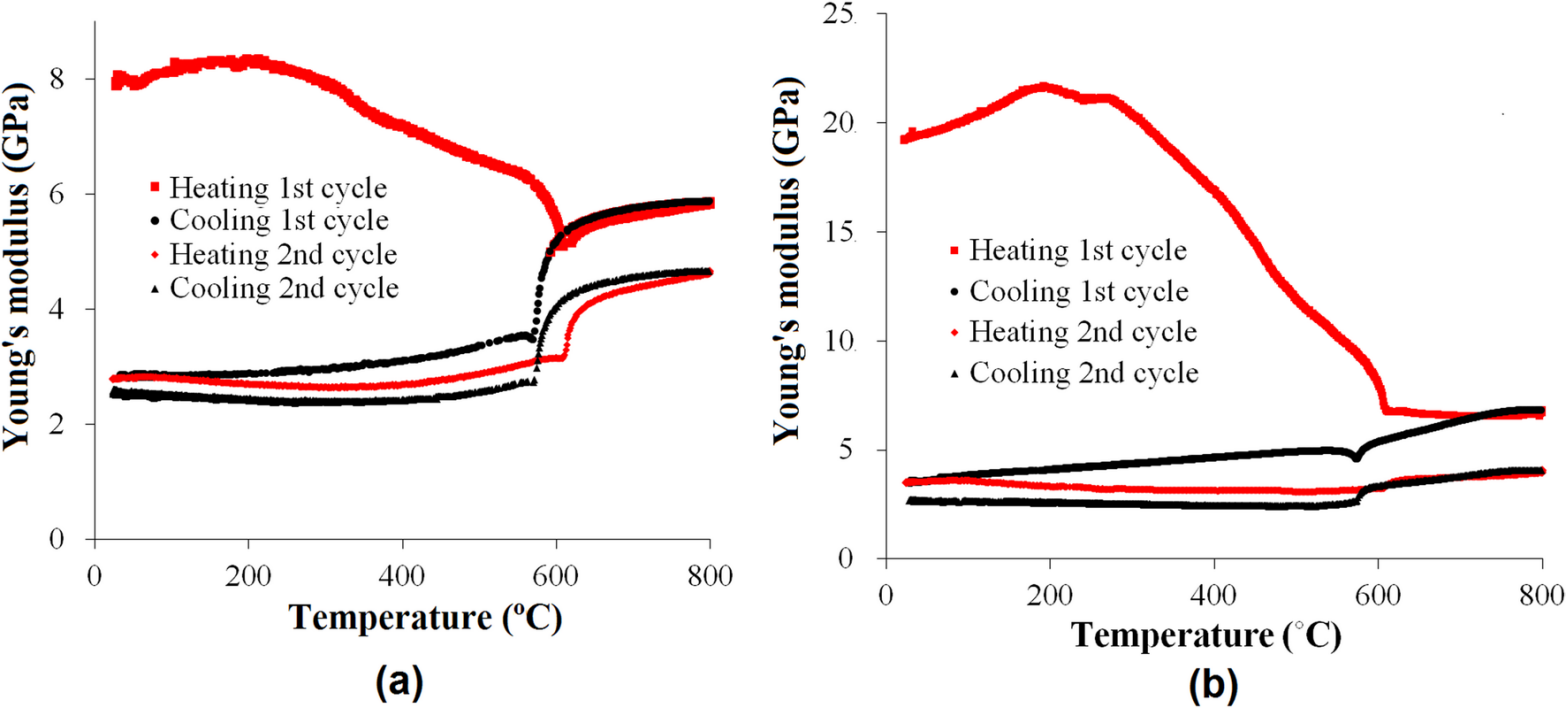
The changes of Young’s modulus with temperature. (**a**) Yellow sandstone, (**b**) Red sandstone.

Figure 2 shows the modulus changes of yellow and red sandstones with temperature during two continuous heating–cooling cycles (peak temperature 800 °C). It can be seen that the initial modulus of the yellow sandstone is around 8 GPa, while that of the red sandstone is around 20 GPa. The difference in Young’s modulus of these sandstones is due to their differences in mineral composition, porosity and crack density. The modulus changes of the yellow sandstone during heating can be seen in four stages. The modulus first increases slightly from room temperature to 250 °C. It then decreases steadily until reaching 500 °C. In the range of 500 °C to 600 °C, the modulus drops quickly. Above 600 °C, however, it increases again yet dramatically. During cooling, the modulus decrease is first minor, but then becomes dramatically when the temperature approaches 600 °C. After that, the change turns out to be very small again. Unlike the changes in the heating stage of the first heating–cooling, in the second heating–cooling cycle, modulus first decreases slightly, but then shows a dramatic increase when the temperature approaches 600 °C. The change of modulus during the cooling stage of the second heating–cooling cycle is similar to the first cycle. It is noted that the critical temperature with dramatic modulus changes in the heating process is higher than that in the cooling stage.

Similarly, the modulus change of the red sandstone in the heating process contains four stages as well. Different from those of the yellow sandstone described above, however, the initial increase (first stage) and the consequent decrease (second stage) of modulus of the red sandstone are much more significant. Moreover, compared with the dramatic modulus increase of the yellow sandstone near 600 °C, the modulus change of the red sandstone is very small. Obviously, the modulus variations revealed above indicate that the rock materials in the heating–cooling cycles have experienced rather complicated deterioration processes that cannot be explained by post-test characterizations.

**Figure 3:**
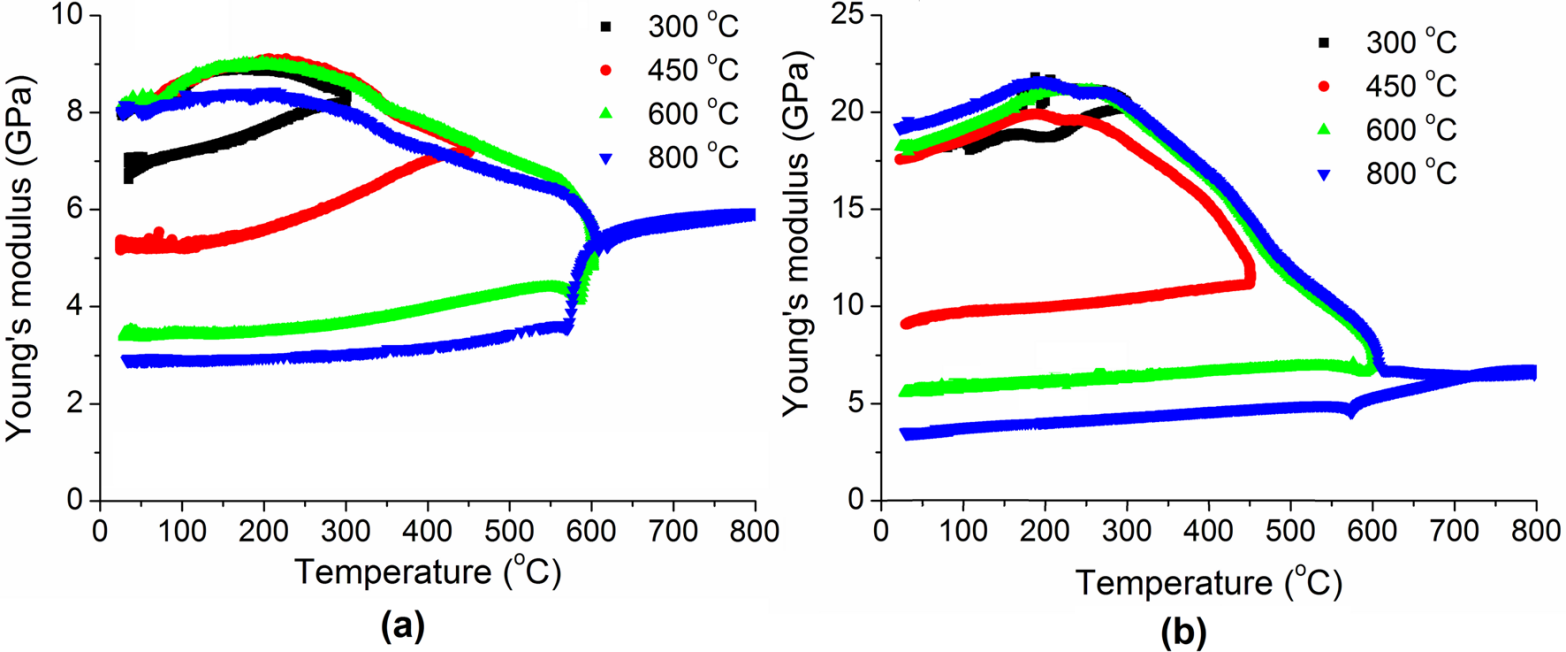
The changes of Young’s modulus with different peak temperatures. (**a**) Yellow sandstone, (**b**) Red sandstone.

To further clarify the deterioration of the sandstones, we carried out the tests with different peak temperatures, as shown in Fig. 3. The modulus difference at the beginning (room temperature) may be due to the different initial structure defects in the specimens. In the heating stage, the changes of modulus up to the different peak temperatures are similar with each other. In the cooling stage, dramatic drop of Young’s modulus appears when the peak temperature is 600 ˚C and beyond. A short increase and a slight decrease then take place after the dramatic drop. In the cases of peak temperatures below 600 ˚C, the moduli decrease smoothly from peak temperatures to room temperature during cooling, particularly for the yellow sandstone. It is interesting to note that the modulus of the yellow sandstone decreases significantly in both the heating and cooling processes; while that of the red sandstone occurs mainly in the heating process.

**Figure 4:**
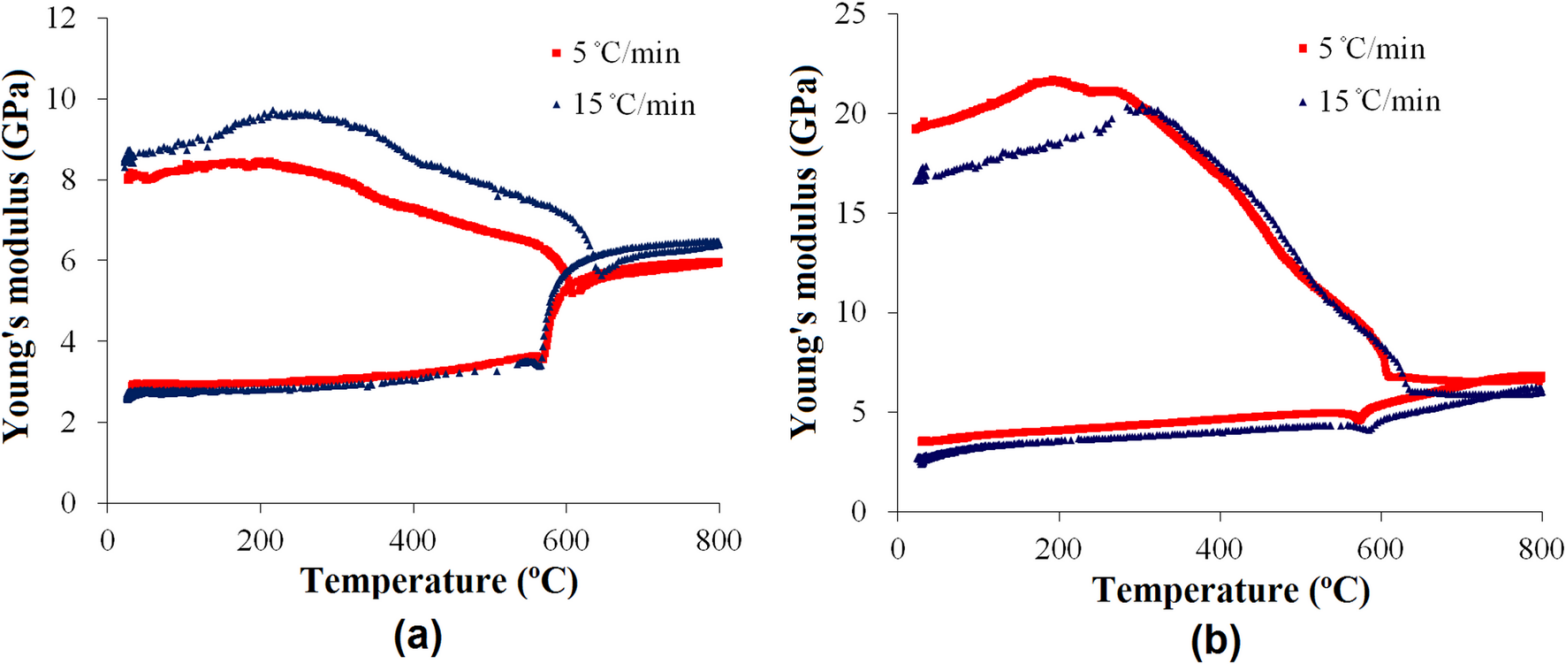
The changes of Young’s modulus with temperature at different heating cooling rates. (**a**) Yellow sandstone, (**b**) Red sandstone.

In a real fire, the heating rate can be up to 20 ˚C /min^21,30^. Moreover, when a fire rescue is conducted, the cooling rate could be very high as well. Therefore, it is very important to clarify the effect of high heating–cooling rate on the rock decay. Thus we carried out the tests at the heating–cooling rate to 15 °C/min (the maximum rate doable in our lab configuration). Figure 4 shows the modulus changes of the two sandstones at different heating–cooling rates. It can be seen that the rate does not affect the basic trend of the modulus change. The modulus difference in the heating stage is mainly due to the different initial status of the specimens (crack density and distribution etc.). At 15 °C/min, during heating the dramatic modulus drop and increase take place at a higher temperature than that at 5 °C/min. This is because the heat conductivities of rocks are very small, leading to temperature gradient and change delay in the rocks.

## Discussion

A rock is a natural aggregate of minerals with internal defects (cracks and pores). Therefore, its elastic modulus is determined by both the mineral components and internal defects. Due to the anharmonic atomic vibration^31^ and phase transformation^32^, the moduli of minerals will change with temperature. Furthermore, in a heating–cooling cycle thermal expansion/shrinkage-induced stress concentration will trigger and enhance the growth of internal cracks. All these factors can affect the microstructures and properties of the rocks and thus lead to the complicated variations of modulus with temperature as observed in Figs. 2, 3, 4.

### The modulus changes of major minerals with temperature

**Figure 5:**
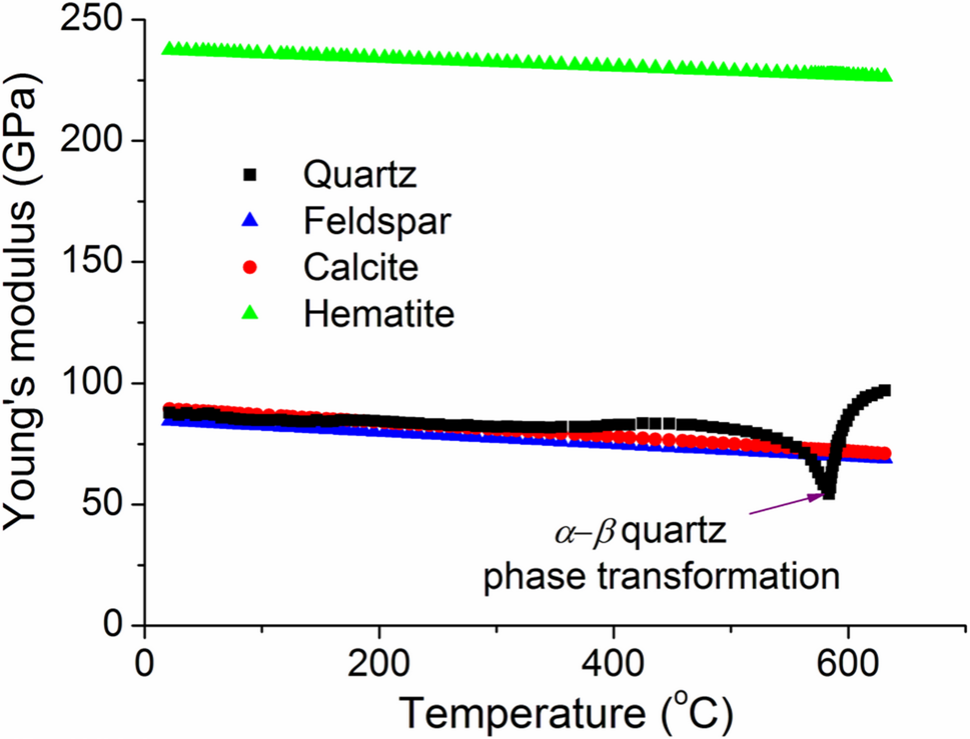
The changes of Young’s modulus of minerals with temperature.

The modulus changes of the major minerals in sandstones with temperature are summarized in Fig. 5. Quartz is the most important component in sandstones. Some studies have been carried out on the modulus changes of quartz with temperature, including both single crystalline^32,33^ and polycrystalline^32,34^. Considering that the distribution of quartz grain in rocks is random, here we only focus on the modulus changes of polycrystalline quartz^32^. As shown in Fig. 5, the Young’s modulus of polycrystalline quartz decrease slightly with temperature from 25 to 520 ˚C, and then further decrease quickly towards a minimum point, followed by a dramatic increase near 573 ˚C. The steady decrease of elastic constants in the low-temperatures is attributed to the atomic force-constant softening^35^. The dramatic change of modulus near 573 ˚C is due to the α – β phase transformation of quartz^32–34^. This has been proved to be energetically dominant and can be explained in the framework of Landau theory^34^. Apparently, this transition should be the main reason for the dramatic modulus changes of rocks near 600 ˚C. It is noted that the critical transition temperature can be affected by pressure, which may explain the difference in the critical transition temperatures^34^.

Feldspars (KAlSi3O8–NaAlSi3O8–CaAl2Si2O8) are a group of rock-forming tectosilicate minerals. Different from quartz, the structure of feldspar are very stable even at 1,000 ˚C^36^. Therefore, its modulus only decreases slightly with temperature due to anharmonic atomic vibration^31,37^. The modulus-temperature relationship of Feldspars can be expressed by a linear function^38^:

1 $$E\left( {{\text{GPa}}} \right) = { 85}{-}0.0{3}(T - {25})$$

where 85 GPa is the averaged Young’s modulus of Feldspars at room temperature. Calcite is also very stable below 700 ˚C. It was reported that ^39^ the changes of the bulk modulus and shear modulus of calcite can be expressed as *K* (GPa) = 79.57–0.023(*T*-25), *G* (GPa) = 32.23–0.009/(*T *− 25), respectively. According to the classic elastic relationship, the Young’s modulus changes of calcite can then be calculated (see Fig. 5). At a high temperature above 700 ˚C, however, calcite will break down via the reaction CaCO_3_ → CaO + CO_2_^39^. Hematite is the mineral component making the sandstone red. This mineral is very stable up to 1,200 ˚C^40^, and therefore its modulus also has a linear relationship with temperature as shown in Fig. 5^31^. Compared with the other mineral components, the modulus of clay is much smaller (~ 6.2 GPa^41^) due to the internal defects. During a heating–cooling cycle, the nucleation of crack occurs in the minerals. Therefore, in the following analysis we assume that the modulus of clay is constant during heating–cooling cycle, and the effect of internal defects will be studied separately.

### The averaged mineral modulus of rocks

**Figure 6:**
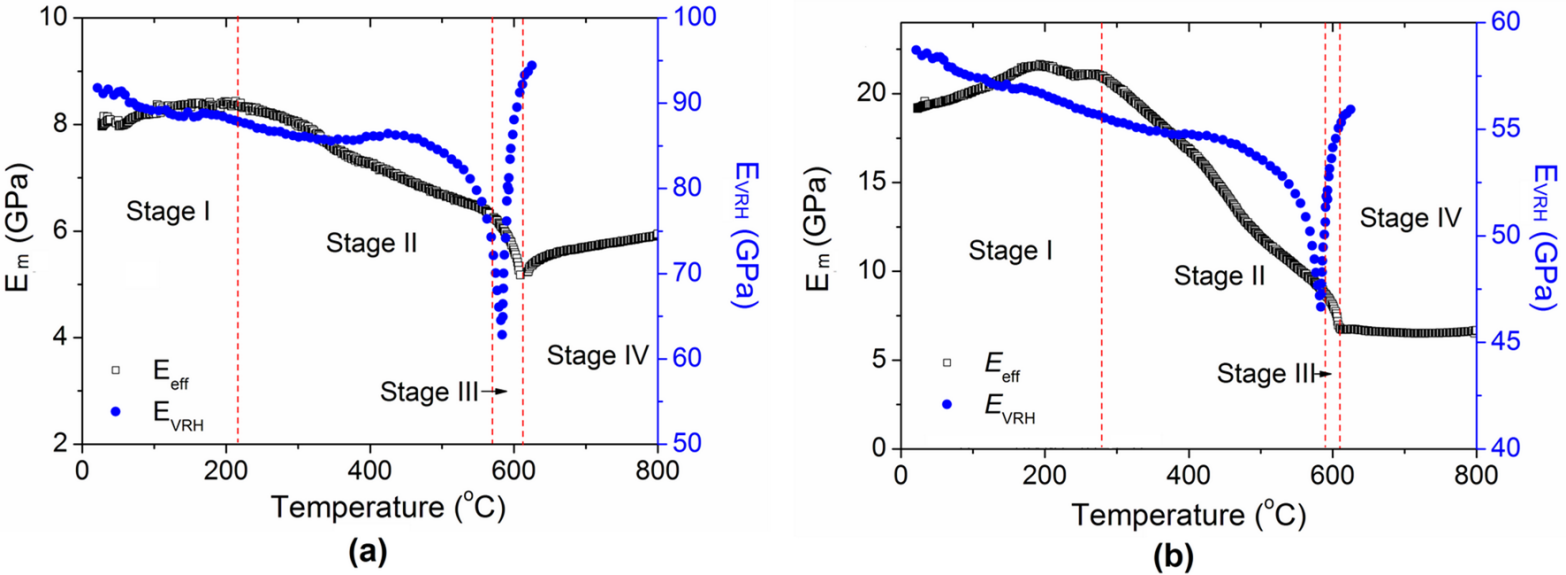
Changes of the averaged mineral modulus (*E*_VRH_) and measured modulus (*E*_m_) during heating. (**a**) Yellow sandstone, (**b**) Red sandstone.

Unlike igneous rocks which have interlocking crystal grains, sandstones contain loosely-coupled mineral grains embedded in clay^2,3^. Assuming that the mineral grains are randomly distributed, the average modulus of minerals can then be estimated by the Voigt-Reuss-Hill (VRH) average method^42,43^, i.e.,

2 $$E_{VRH} = {{(E_{eff}^{v} + E_{eff}^{R} )} \mathord{\left/ {\vphantom {{(E_{eff}^{v} + E_{eff}^{R} )} 2}} \right. \kern-\nulldelimiterspace} 2}$$

where $E_{eff}^{v} = \sum\limits_{i}^{N} {f_{i} E_{i} }$ is the effective Voigt modulus and $E_{eff}^{R} = {1 \mathord{\left/ {\vphantom {1 {\sum\limits_{i}^{N} {{{(f_{i} } \mathord{\left/ {\vphantom {{(f_{i} } {E_{i} }}} \right. \kern-\nulldelimiterspace} {E_{i} }}} )}}} \right. \kern-\nulldelimiterspace} {\sum\limits_{i}^{N} {{{(f_{i} } \mathord{\left/ {\vphantom {{(f_{i} } {E_{i} }}} \right. \kern-\nulldelimiterspace} {E_{i} }}} )}}$ is the effective Reuss modulus; *f*_i_ and *E*_i_ are the volume fraction and modulus of the *i*th mineral component, respectively. This VRH average method has been widely and successfully applied in evaluating the modulus of rocks and other composite materials^42,43^. According to Eq. (2), one can get the average mineral modulus of yellow and red sandstones and their changes with temperature as shown in Fig. 6a, b, respectively. For comparison, the measured modulus (*E*_m_) during the first heating stage is also added in the figures.

It is clear that below 300 ˚C, for both cases the average moduli of minerals decrease with temperature, which is in contrast with the initial increase of the measured modulus *E*_m_. Moreover, although the red sandstone has similar mineral components to those of the yellow sandstone, the decrease of *E*_m_ of the former during the heating stage is much larger than that of the latter. In the neighborhood of 600 ˚C, the sudden decrease and increase of *E*_m_ is apparently the effect of α − β quartz transition. However, the basic trends of two sandstones are very different. More importantly, the modulus changes of the mineral components are reversible, which also contrast to that of rocks. Therefore, to explain the experimental result, we must consider the evolution of internal cracks in rocks as well.

### Crack evolution during heating–cooling

In rocks, three types of microcracks can be distinguished^23^: (1) intergranular microcracks that close to grain boundaries, (2) intragranular microcracks that emanate from a pore or a grain boundary, and (3) transgranular microcracks that run across one or several grains. However, it is very difficult to carry out direct and independent measurements of crack density such as from scanning electron microscope^44^. It is even much more difficult to accurately characterize the evolution of cracks during the heating–cooling process.

According to the theory of damage mechanics^22,23^, the effective modulus of a solid with micro-cracks can be described by

3 $$E(T) = {{E_{0} (T)} \mathord{\left/ {\vphantom {{E_{0} (T)} {(1 + {{16(1 - v_{0}^{2} )(1 - {{3v_{0} } \mathord{\left/ {\vphantom {{3v_{0} } {10}}} \right. \kern-\nulldelimiterspace} {10}})} \mathord{\left/ {\vphantom {{16(1 - v_{0}^{2} )(1 - {{3v_{0} } \mathord{\left/ {\vphantom {{3v_{0} } {10}}} \right. \kern-\nulldelimiterspace} {10}})} {9\rho (1 - v_{0} /2}}} \right. \kern-\nulldelimiterspace} {9\rho (1 - v_{0} /2}})}}} \right. \kern-\nulldelimiterspace} {(1 + {{16(1 - v_{0}^{2} )(1 - {{3v_{0} } \mathord{\left/ {\vphantom {{3v_{0} } {10}}} \right. \kern-\nulldelimiterspace} {10}})} \mathord{\left/ {\vphantom {{16(1 - v_{0}^{2} )(1 - {{3v_{0} } \mathord{\left/ {\vphantom {{3v_{0} } {10}}} \right. \kern-\nulldelimiterspace} {10}})} {9\rho (1 - v_{0} /2}}} \right. \kern-\nulldelimiterspace} {9\rho (1 - v_{0} /2}})}}$$

where υ_0_ is the averaged Poisson’s ratio of matrix, and $\rho$ is the crack density. The crack density can be expressed as $\rho = {{\sum\limits_{m = 1}^{N} {a^{(m)3} } } \mathord{\left/ {\vphantom {{\sum\limits_{m = 1}^{N} {a^{(m)3} } } V}} \right. \kern-\nulldelimiterspace} V}$, where *a*^(m)^ is the dimension of the *m*-th crack and *V* is the specimen volume^22^. Considering that the changes of Poisson’s ratio of mineral components with temperature are very small, the variation rate of crack density against temperature can be expressed as:

4 $$\frac{d\rho }{{dT}} \propto \frac{{d({{E_{VRH} } \mathord{\left/ {\vphantom {{E_{VRH} } {E_{m} }}} \right. \kern-\nulldelimiterspace} {E_{m} }})}}{dT}$$

Apparently, the changes of crack density are proportional to the changes of *E*_VRH_/*E*_m_. Using this equation we can qualitatively analyze the evolution of crack density during a heating–cooling cycle.

As shown in Fig. 6a, b, in Stage I for both cases the average modulus of mineral components *E*_VRH_ decreases while the measured modulus *E*_m_ increases. That is, in this stage *E*_VRH_/*E*_m_ decreases with temperature and thus the crack density should decrease as well. This can be attributed to the temporary closure of cracks. In rocks, most microcracks appear in the grain boundaries of mineral grains. The width of a crack is normally in the order of several micrometers and the grain size ranges from several hundred micrometers to few millimeters^44^. Considering that the coefficient of thermal expansion of minerals is in the order of 1 × 10^–5^ 1/K^45^, the thermal expansion of quartz, feldspar and calcites grains can push the surrounding microcracks to close.

In stage II, *E*_m_ starts to decrease with a higher rate than that of *E*_VRH_. Therefore, *E*_VRH_/*E*_m_ and the crack density increase accordingly. The decrease of *E*_m_ should be attributed to the formation and propagation of new cracks. In this stage, the continuous thermal expansion of mineral grains makes them contact and push each other. Due to the non-uniform shape and constraints, some grains will be pushed to move or rotate, creating new cracks around the grains. The higher the temperature, the more new cracks and voids are created, leading to the continuous decrease of Young’s modulus.

In stage III, the measured modulus decreases dramatically, corresponding to the dramatic modulus decrease of quartz. It is noted that in the yellow sandstone from 500 ˚C to that of the minimum point, *E*_VRH_/*E*_eff_ changes from 13.3 to 11.15, indicating that the crack density is decreasing slightly. However, in the red sandstone, the ratio change is difficult to be determined. We can only conclude that the crack density change is not significant in this stage.

After the transition point, i.e., in stage IV, ratio *E*_VRH_/*E*_m_ of the yellow sandstone changes from 11.15 to 18.33, indicating a dramatic increase of crack density. This is because the volume of β quartz is much larger than that of α quartz, creating more cracks during the phase transition. *E*_VRH_ of the red sandstone increases dramatically, while *E*_m_ remains almost the same, and thus its crack density should also increase in this stage. Overall, the modulus changes in stage IV should be a balanced result of quartz hardening and crack nucleation softening.

Similar analysis can be conducted to understand the crack evolution during cooling and in the second heating–cooling cycle. As shown in Fig. 2a, in the first cooling process, the modulus of the yellow sandstone decreases slightly at high temperature and then drops very quickly around 600 ˚C, following the modulus change of quartz. Ratio *E*_VRH_/*E*_m_ does not change too much in the process, indicating that crack density remains almost unchanged in the process. Hence, the sudden decrease of *E*_m_ is mainly due to the modulus decrease of quartz. The same conclusion can be made on the red sandstone. Since the crack density in the red sandstone generated in the heating process is much higher than that in the yellow sandstone, the effect of quartz phase transformation in the former is much smaller than that in the latter. After then, *E*_VRH_ keeps increasing but the change of *E*_m_ is very small in both of the sandstones. Therefore, ratio *E*_VRH_/*E*_m_ and crack density should increase in this stage. Considering that during the cooling stage all the grains are shrinking_,_ the increase of crack density should be attributed to the opening of existing cracks that were closed by thermal expansion stress. The decrease of modulus observed in the test with other peak temperatures should be due to the same reason of crack opening.

Since many cracks have been generated in the first heating–cooling cycle, the nucleation of new cracks during the second cycle is very small. At the beginning of heating, the thermal expansions of mineral grains are not enough to close the cracks. *E*_m_ increases again in the yellow sandstone only when a large expansion is produced by the α − β quartz transition. In the second cooling process, the trend is almost reversible, indicating that a few new cracks are generated in the process.

### Young’s modulus measured under different conditions

**Figure 7:**
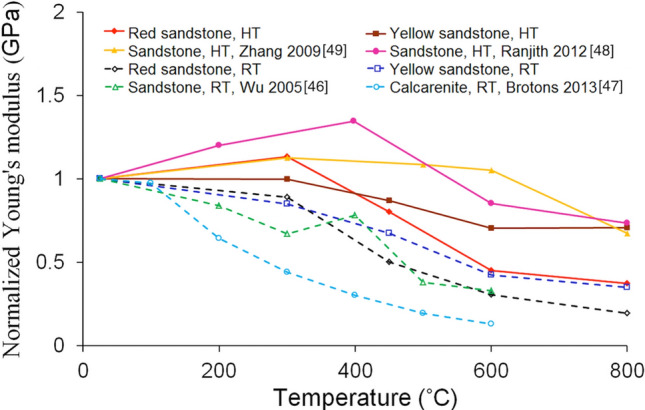
The modulus changes of rocks with temperature under different conditions. (HT: modulus measured at high temperature, RT: modulus measured at room temperature after heat treatment.)

Variations of rock modulus with temperature in the literature are not consistent with each other^46–49^. This is because different techniques and rocks were used in measuring the modulus. As shown in Fig. 7, the reported modulus can be divided into two categories, measured (1) at high temperature and (2) at room temperature after heat treatment. Our result clearly shows that the moduli of rocks measured at the peak temperature are completely different from those measured at room temperature after heat treatment. To clarify this further, we summarized the modulus changes with different peak heating temperatures in Fig. 7. For the convenience of comparison, all the moduli in the figure were normalized by the corresponding moduli at room temperature. Although the rock types are different, it is clear that the moduli measured at high temperature are larger than those measured after heat treatment. Moreover, at a temperature below 300 ˚C the measured moduli at high temperature increase slightly due to the closure of cracks, while the moduli measured after heat treatment always decrease. Therefore, in discussing the temperature effect on the rock decay, it is very important to differentiate the experimental results obtained by different techniques, because the microstructure of rocks could be significantly changed during the cooling process.

## Conclusions

This paper has presented a comprehensive investigation into the decay of red and yellow sandstones in heating–cooling cycles by instantly monitoring their modulus changes. In the heating stage the modulus changes of both rocks can be divided into four stages, i.e. stage I: the initial slightly increasing from room temperature to ~ 300 ˚C, stage II: the decrease stage from 300 to 500 ˚C, stage III: the dramatic decrease from 500 to 600 ˚C, and stage IV: above the critical temperature near 600 ˚C. In the cooling stage, the changes of modulus consist of two stages divided by the critical temperature. In the second cycle, significant modulus changes only occur near the critical temperature of the α − β phase transformation of quartz. Changing the heating–cooling rate doesn’t affect the basic trend. The modulus changes are mainly attributed to the internal cracks evolutions and α − β phase transformation of quartz. According to the theory of composite material and damage mechanics, a relationship between the measured modulus and the crack density was established, which can be straightforwardly used for revealing the decay mechanisms of rocks during heating–cooling cycles.

## Methods

Two rocks, yellow sandstone and red sandstone from Xuzhou, China, are studied in this paper. The mineralogical composition of sandstones was identified by performing powder X-ray diffraction (XRD) analysis. The yellow sandstone mainly consists of quartz (66.5, vol.%), feldspar (27, vol.%), calcite (5, vol.%), and clay minerals (1.5, vol.%). The main compositions of the red sandstone are quartz (39, vol%), feldspar (27, vol.%), calcite (16, vol.%), hematite (5, vol.%) and clay minerals (13, vol%). All the specimens with a dimension of 40 × 4 × 10 mm (± 0.1 mm) were cut from large block rock materials (visibly free of fractures). The surfaces of the specimens were carefully polished by abrasive paper (up to #800).

The specimen is suspended by supports in the furnace (see Fig. 1), in which heating–cooling cycles with different peak temperatures and rates were conducted (see Table 1). At the peak temperature, a holding time of 10 min was set for all tests. In the cases with a peak temperature of 800 ˚C, the second heating–cooling cycle was also conducted. During the heating–cooling cycles, the specimen was excited by the impact bar every 15 s. The vibration signal of the specimen was collected by a ceramic hollow bar and recorded by a high-precision microphone outside the furnace. The Young’s modulus can be calculated by the following equation^26,28^

5 $$E = 0.9465 \times (mf^{2} ) \times (\frac{{L^{3} }}{{mh^{3} }})$$

where $m$ the mass of the bar, $f$ the fundamental flexural resonant frequency of the bar, $L$ is length, $w$ is width, $h$ is thickness. For each heating/cooling cycle, repeated tests have been carried out for at least three times.

**Table 1:** The details of impulse excitation technique tests.

Sandstone type	Peak temperature	Heating–cooling rate (°C/min)
Yellow	800	5
	800	20
	600	5
	450	5
	300	5
Red	800	5
	800	20
	600	5
	450	5
	300	5

Considering that the mass and dimension of specimen would change during heating–cooling cycles, all the measured modulus has been amended accordingly via Eq. (5). After obtaining the evolution of Young’s modulus with temperature, a mean field model will be established to analyze the crack density changes of sandstone during the heating–cooling cycles.

## Data Availability

The data used to support the findings of this study are included within the article.
